# Ultra late onset group B streptococcal sepsis with acute renal failure in a child with urethral obstruction: a case report

**DOI:** 10.1186/1752-1947-6-68

**Published:** 2012-02-20

**Authors:** Daniela Freudenstein, Konrad Reinshagen, Angela Petzold, Angelika Debus, Horst Schroten, Tobias Tenenbaum

**Affiliations:** 1 University Children's Hospital Mannheim, Heidelberg University, Theodor-Kutzer-Ufer 1-3, 68167 Mannheim Germany; 2 Department of Pediatric Surgery, University Hospital Mannheim, Heidelberg University, Germany; 3 Institute for Medical Microbiology and Hygiene, Medical Faculty Mannheim, Heidelberg University, Germany; 4 Department of Clinical Radiology and Nuclear Medicine, Medical Faculty Mannheim, Heidelberg University, Germany; 5 Department of Pediatric Surgery, University Hospital Hamburg-Eppendorf, Hamburg, Germany

**Keywords:** Children, urinary tract infection, GBS, late onset sepsis

## Abstract

**Introduction:**

Group B streptococci are a well-known cause of early and late onset sepsis. In neonates and older children gram-negative bacteria are mostly found in urinary tract infections and urosepsis. In adults predisposing factors for group B streptococci urinary tract infection may include diabetes mellitus and chronic renal failure.

**Case presentation:**

We present a rare case of a five-month-old Caucasian boy with ultra late onset urosepsis and acute renal failure caused by group B streptococci serotype V. Excretion urography showed a subvesical obstruction that consequently was surgically corrected after antibiotic treatment of the acute infection.

**Conclusions:**

Group B streptococci serotype V, urogenitary tract malformations, previous hospitalization and medical interventions may be important risk factors for the development of ultra late onset Group B streptococci sepsis in non-neonates.

## Introduction

Group B *Streptococcus *(GBS) is a leading cause of infection in newborns, pregnant women, and older persons with chronic medical illness. Cervicovaginal colonization with GBS in pregnant women can result in vertical transmission of GBS to neonates, with a limited number of GBS capsular serotypes being disproportionately associated with colonization and disease; serotypes Ia, III, and V, for example, cause the majority of invasive infections in older persons [1]. Multiple serotypes of GBS also cause urinary tract infections (UTIs), which encompass asymptomatic bacteriuria, cystitis, pyelonephritis, urethritis, and urosepsis [1,2]. GBS asymptomatic bacteriuria is particularly common among pregnant women; however, those most at risk for cystitis due to GBS are older persons and immunocompromised individuals [2,3]. Predisposing factors for GBS UTI may include diabetes mellitus and chronic renal failure [4].

## Case presentation

**Figure 1:**
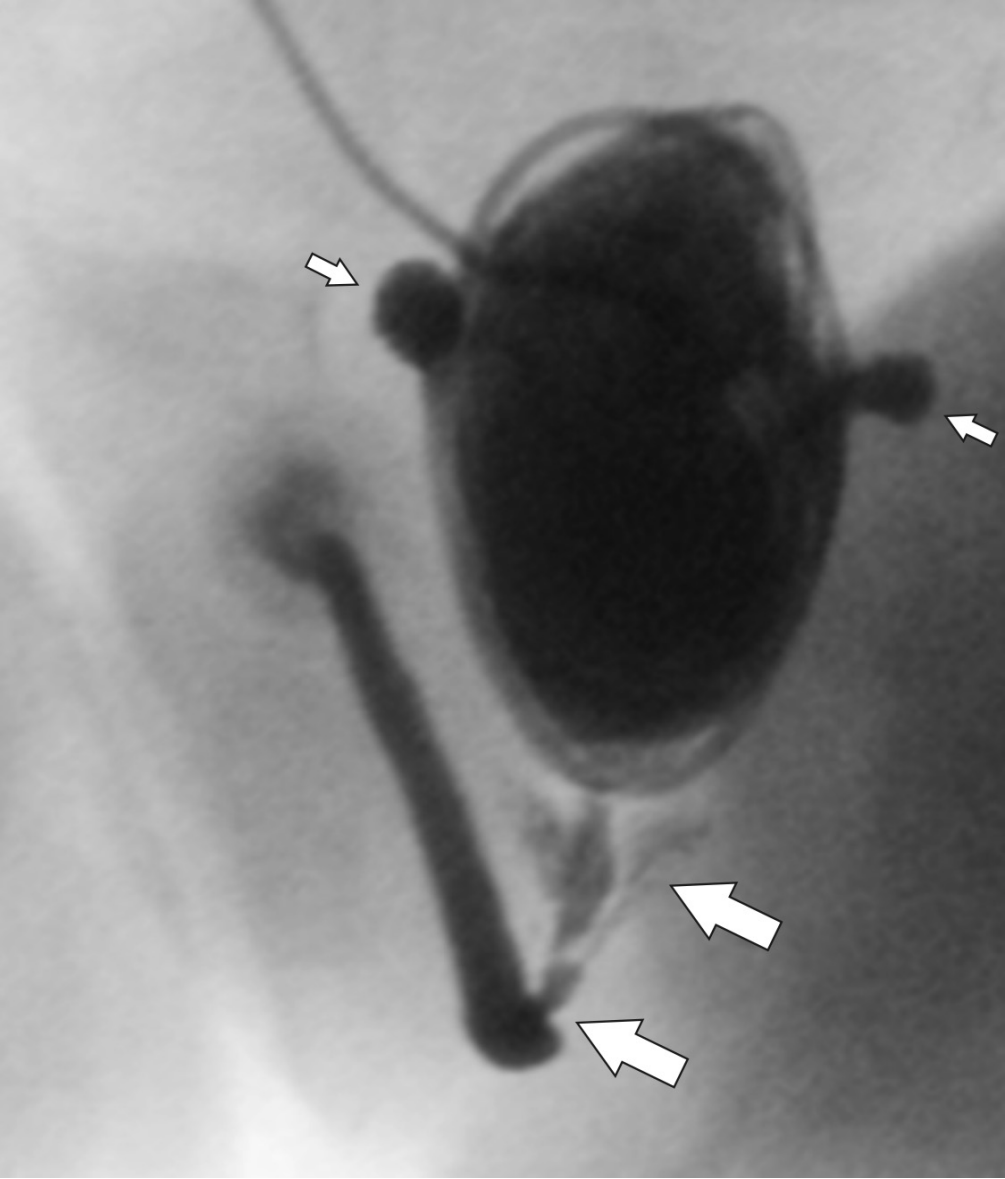
**The voiding cystourethrosonography shows a urethral obstruction with a pre-stenotic dilatation of the urethra and the seminal vesicles (thick arrows)**. The patient also had a thickened bladder and two bladder diverticuli (small arrows).

A critically ill five-month-old Caucasian boy presented with a four-day history of fever of unknown origin up to 39°C at our hospital. One week prior to the current admission he had undergone herniotomy and orchidopexy and was discharged after two days. During this hospital admission a distended bladder had been observed after herniotomy and the child was subsequently catheterized. The catheterization was described as uneventful; therefore, no further diagnostics were initiated. On the current admission, the child appeared septic without inflammation of his surgical wounds. Blood samples revealed a leucoytosis of 31, 390/μL with 61% neutrophils, hemoglobin 6.60 g/dL, platelet count 584, 000/μL, C-reactive protein (CRP) 243.1 mg/L, procalcitonin 6.16 μg/L, creatinine 3.28 mg/dL, hydroxyurea 127 mg/dL and potassium 6.01 mmol/L. Mid stream urine showed 250 leukocytes/μL and mild erythrocyturia. An abdominal ultrasound showed a hydronephrosis grade three and reduced differentiation of cortex and medulla of both kidneys as a sign of pyelonephritis as well as a bladder wall hypertrophy. He was hospitalized with the tentative diagnosis of urosepsis accompanied by acute renal failure. Microbiologic testing revealed GBS and *Enterococcus faecalis*, both in the blood culture and in the urine. Further serological testing identified GBS serotype V. Antibiotic therapy with ampicillin and ceftriaxone was given for 14 days intravenously. Since transurethral catheterization failed a suprapubic bladder catheter was implanted to relieve hydronephrosis and infusion therapy was mantained. Due to severe anemia, a red blood cell transfusion was also performed. During the further clinical course blood and urine values normalized. Excretion urography revealed a subvesical obstruction (Figure 1) and subsequently he underwent a surgical correction.

## Discussion

GBS infections in children have diverse clinical characteristics in patients with different ages at disease onset [5,6]. While early-onset GBS sepsis is a well known cause of neonatal sepsis and meningitis, little information is available on GBS disease in children older than three months. Risk factors for sepsis caused by GBS in neonates are prematurity and preterm rupture of membranes. In non-newborns, babies with cardiovascular or urogenital abnormalities, vascular disease, neurological or immune deficiency are also prone to GBS infections [5,6]. Here, we report on a five-month-old baby with an ultra-late onset urosepsis caused by GBS serotype V. In the literature, urinary tract infection and acute tonsillitis have rarely been described to be the clinical manifestation of GBS infection in children older than three months of age in Taiwan [7]. Most cases of early and late onset sepsis including meningitis caused by GBS were caused by serotype III, followed by serotype Ia. Serotype V, which was isolated in our patient, was ranked third (6% of total isolates) in children with late onset sepsis [8]. In adults, serotype V has been demonstrated to be the most common GBS serotype in urinary tract infections [3]. Of note, a recent epidemiological study demonstrated a major impact of premature birth on ultra-late GBS meningitis in young babies [9]. Children presenting with ultra-late onset sepsis, may be susceptible to GBS infection due to an underlying condition such as urogenital malformations. Apart from the colonization of the mothers, hospitalization and medical interventions may also be an important risk factor for the development of ultra-late onset sepsis [3,5,6]. In our case, the patient's mother tested GBS negative in a vaginal smear. Instead, iatrogenic GBS infection might have occurred after the recent hospitalization, where the child underwent herniotomy, orchidopexy, and bladder catheterization.

## Conclusions

GBS may be a more common uropathogen in children than previously recognized. GBS serotype V, urogenitary tract malformations, previous hospitalization and medical interventions may be important risk factors for the development of ultra-late onset GBS sepsis in non-neonates. Prompt institution of therapy with antibiotics active against GBS following sensitivity studies could prevent systemic septic complications in this group of frail patients. Clearly, further studies are required to characterize the impact on pathogenicity and virulence of GBS in children and the epidemiology of such micro-organisms.

## Consent

Written informed consent was obtained from the patient's next-of-kin for publication of this case report and any accompanying images. A copy of the written consent is available for review by the Editor-in-Chief of this journal.

## Competing interests

The authors declare that they have no competing interests.

## Authors' contributions

DF, KR AP, AD, HS, TT and FC analyzed and interpreted the patient data. AP performed the microbiological examination, AD the radiological examination and interpretation, DF, KR, HS, and TT were major contributors in writing the manuscript. All authors read and approved the final manuscript
